# Characterization and Discrimination of Italian Olive (*Olea europaea sativa*) Cultivars by Production Area Using Different Analytical Methods Combined with Chemometric Analysis

**DOI:** 10.3390/foods11081085

**Published:** 2022-04-09

**Authors:** Emilia Pucci, Domenico Palumbo, Adriana Puiu, Antonia Lai, Luca Fiorani, Claudia Zoani

**Affiliations:** 1 Biotechnology and Agroindustry Division (SSPT-BIOAG), Department for Sustainability, Italian National Agency for New Technologies, Energy and Sustainable Economic Development, Casaccia Research Centre, Via Anguillarese 301, 00123 Roma, Italy; claudia.zoani@enea.it; 2 Resource Efficiency Division (SSPT-USER), Department for Sustainability, Italian National Agency for New Technologies, Energy and Sustainable Economic Development, Casaccia Research Centre, Via Anguillarese 301, 00123 Roma, Italy; domenico.palumbo@enea.it; 3 Technology Applications for Security, Health and Heritage Division (FSN-TECFIS), Fusion and Technology for Nuclear Safety and Security Department, Italian National Agency for New Technologies, Energy and Sustainable Economic Development, Frascati Research Centre, Via Enrico Fermi 45, 00044 Frascati, Italy; adriana.puiu@enea.it (A.P.); antonia.lai@enea.it (A.L.); luca.fiorani@enea.it (L.F.)

**Keywords:** *Olea europaea* L., cultivars, trace element profile, geographical origin, ICP-AES, ICP-MS, LPAS, chemometrics

## Abstract

Olives and olive products are particularly important for the national agroindustrial sector, for the aspects related to the production territory (authenticity), and for the link with the Mediterranean Diet. Several studies indicate that the elemental profile of olive and olive products depends on the production area in which the olive trees were grown, and the elemental content of the olives can be used as a marker of the production area. In order to confirm this hypothesis, the multi-elemental profile of olive drupes and olive leaves of eleven cultivars arising from two different production areas was evaluated through ICP-MS and ICP-AES techniques. In addition, some leaf samples were analysed by LPAS in order to evaluate the applicability of this new analytical technique for determining the geographic origin. The obtained results, combined with chemometric tools, showed the possibility of discriminating samples according to the production area on the basis of the elemental content, as well as by LPAS.

## 1. Introduction

The olive tree (*Olea europaea* L.) is an evergreen tree cultivated for thousands of years throughout the Mediterranean basin, which represents the largest international olive-growing area [1,2]. The European Union holds the record in terms of olives and olive oils production (69% of the world production), exportation, and consumption. Spain (63%), Italy (17%), Greece (14%), and Portugal (5%) cover 99% of the olive oil production in Europe [3].

In addition to their economic value, olive products such as table olives and olive oil are the basis of the Mediterranean Diet [4] and have a high nutritional and medicinal value. Their consumption was found to be a protective factor against heart disease, some cancers, diabetes, and inflammatory and autoimmune diseases, thanks to the high levels of monounsaturated fatty acids, phenolic compounds, and antioxidants that they contain [2]. Olive by-products have a high value, too; for example, the olive leaf extract is characterised by a high content of polyphenols with antioxidant and antimicrobial activity, which enable its use as a food additive [5].

To guarantee the quality, genuineness, and high market value of olive products, the European Union has adopted the system of EU Geographical Indications (PDO—Protected Designation of Origin; PGI—Protected Geographical Indication; and TSG—Traditional Specialty Guaranteed), to designate to those products whose characteristics of quality and typicality are closely linked to the production territory [6,7,8,9].

Several authors have shown that trace elements’ distribution in olives and olive oils depends on the mineral content and elemental bioavailability in the growing soil [6,8,10,11]. Therefore, the geochemical signature of the soil, which depends on the production area, is transferred to the olives [11]. This means that the elemental profile of the olive products depends on the geographical area in which the olive trees were grown, thus potentially allowing to discriminate between products of different geographical origins and even, for example, PDO products from others not considered in this certification, thus giving the possibility to highlight fraud [8,12]. Furthermore, other studies have shown that the elemental profile in olive products is more influenced by the geographical origin of the product than by the cultivar [8,10,11,12].

Several analytical techniques, in combination with multivariate statistical analysis, have been used to identify the geographical origin of olive products. These are based on the determination of the physical–chemical parameters, such as fatty acids, triacylglycerols, and sterols. Other techniques, such as Nuclear Magnetic Resonance (NMR), Fourier Transform Infrared (FT-IR) Spectroscopy, Gas Chromatography–Ion Trap Mass Spectrometry (GC–ITMS), Isotopic Ratios Mass Spectrometry (IRMS), Near Infrared Spectroscopy (NIR), and Mid-Infrared Spectroscopy (MIR) have been applied for olive product analyses studying signals related to specific compounds such as volatile patterns or isotopic ratios [6,10,13,14,15,16,17,18,19,20].

The use of elemental analysis for the geographical traceability of olive products can be an alternative to other approaches. For the trace element analysis in olive products, the most frequently used are Inductively Coupled Plasma Atomic Emission Spectrometry (ICP-AES) and Atomic Absorption Spectrometry (AAS) [19,21,22]. ICP-AES is one of the most widely applied analytical techniques for the determination of trace elements in a wide range of matrices. Its use is preferred over AAS, as it allows a wide range of elements to be analysed simultaneously, significantly reducing the measurement time for each sample. It also provides very low detection limits and a wide range of analytical linearity [23].

Oil and fat food matrices are particularly difficult to analyse for their trace metal contents since some of them are present at very low concentration levels. For this reason, Inductively Coupled Plasma Mass Spectrometry (ICP-MS) is considered an interesting tool because of its well-known high sensitivity (it allows the quantification of elements at a very low concentration level, at the order of ppb and ppt) and its wide linear dynamic range, wide elemental coverage, and high sample throughput [10,24]. In all cases (AAS, ICP-AES, and ICP-MS), sample treatments, such as wet, dry, or (mostly) microwave digestions, are required in order to eliminate the organic matrix since the direct analysis faces many drawbacks and usually causes the extinction of plasma.

A new spectroscopy technology based on photoacoustic effects, named LPAS (Laser Photo Acoustic Spectroscopy), which was developed at the ENEA Diagnostic Metrology Laboratory (Frascati), has been proposed to analyse samples without or negligible manipulations. This technology exploits infrared absorption and operates with a Quantum Cascade Laser (QCL). The laser beam is modulated at an audio frequency and directed into a resonant cell where it hits the investigated sample absorbing the incident radiation. The sample irradiated by the beam generates a rise in temperature in the cell closed volume, thus producing a pressure wave. In general, the sound detection subsystem is made of a microphone connected with a lock-in amplifier synchronized with the modulator. The output signal is proportional to the sample absorption. The experiments are conducted in the “fingerprint region”, a large band of the infrared (IR) spectrum where many organic compounds can be identified. In some sense, LPAS is similar to IR spectroscopy, but with the important advantage of an unrivalled power of the radiation source and very narrow linewidth (laser vs. lamp). Main advantages offered by LPAS are the possibility to perform non-destructive measurements, real time operation, high resolution, and no need for sample manipulation. For these characteristics, LPAS has already been successfully applied both in the horticulture field, to detect pathogen attack, and in post-harvest shelf-life analysis [25,26,27,28], as well as in fraud detection [29].

This study is aimed to evaluate the suitability of the trace element profile in olives of different cultivars as a marker of the production area and the influence of the cultivar itself. For this purpose, the multi-elemental profile of olive drupes and olive leaves of eleven cultivars arising from two different production areas was evaluated. ICP-MS and ICP-AES were applied to determine 14 elements (Sr, Cu, Rb, Ti, Ni, Sn, Cr, V, Co, Sb Cd, Pb, As, and Zr) in olives and 17 elements (Al, Sr, Fe, Ba, Rb, Mn, Zn, Cu, Ti, As, Sb, Pb, Ni, Cd, Co, Cr, and V) in olive leaves. Moreover, with the purpose to verify the suitability of LPAS for geographical origin demonstration, same samples of leaves were analysed, and the results compared with the ones obtained by ICP-AES. Finally, chemometric approaches were applied for extracting the relevant information to discriminate between the different cultivars and the different geographical origins.

## 2. Materials and Methods

### 2.1. Sampling and Sampling Sites

**Figure 1:**
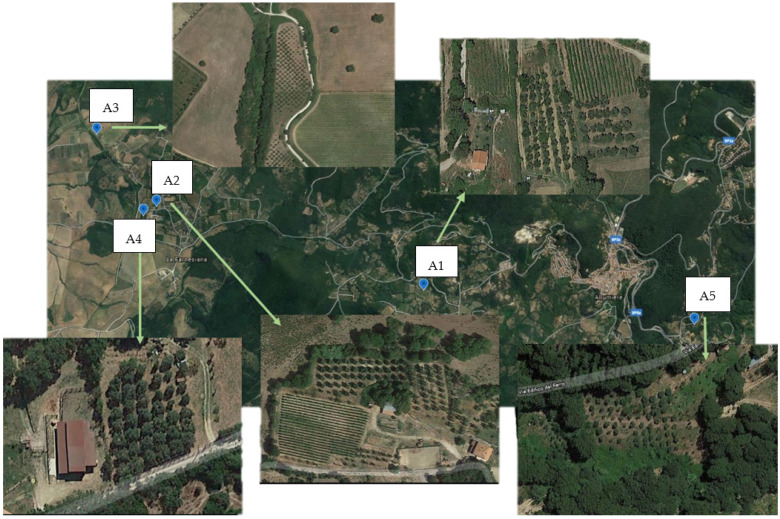
Olive orchards (experimental fields A1, A2, A3, A4, A5) in the municipality of Allumiere.

Samples of drupes and olive leaves were collected in two different Italian production areas, in the Lazio region, in October 2019. The sampling period was selected considering its correspondence with the time when olives are usually harvested in Italy. The following olive orchards were considered for sampling: the orchard hosting the olive trees’ collection of the ENEA Casaccia Research Centre (Rome), and 5 different olive orchards in the municipality of Allumiere (province of Rome). The 5 olive orchards in Allumiere were chosen based on the cultivars hosted and the different locations: the A1 site was located near the Allumiere village north facing; the A2, A3, and A4 sites were located to the north-west of Allumiere in a flat area near the Mignone river; and the A5 site was located in the south of Allumiere in a south-facing hamlet called La Bianca (Figure 1).

The study was carried out considering 11 different olive cultivars: Cipresso, Moraiolo, Canino, Pendolino, Itrana, Frantoio, Uovo di piccione, Maurino, Ascolana, Leccino, and Ortice. The cultivars were selected on the basis of their different commercial use: oil cultivars (Cipresso, Canino, Frantoio, Leccino, Maurino, Moraiolo, and Pendolino), table cultivars (Ascolana, Uovo di piccione), and dual purpose cultivars (Itrana and Ortice).

For each sample, about 500 g of drupes and 100 g of leaves were manually collected around the canopy of each plant (approximately 1.5 m high). Homogeneous plants were chosen for each cultivar in terms of crown conformation and fruit load. The fruits were chosen without evident signs of structural damage, at full ripeness and pigmentation. The leaves were chosen to avoid those with imperfections, such as insect infestation, the presence of honeydew, droppings, or necrosis. Overall, 37 samples of drupes and 37 samples of leaves were collected in the different experimental areas, for a total of 74 samples.

After collection, samples were washed with ultrapure water (resistivity > 15 MΩ cm). Then the olive samples were stored at T = −20 °C, while the leaf samples were dried at room temperature (20 ± 5 °C) for ten days in the dark and under ventilation and then stored in plastic bags at room temperature.

### 2.2. Sample Pre-Treatment

Laboratory samples were subjected to a pre-treatment procedure to obtain representative portions to be used for the different analyses. In particular, the pre-treatment procedure was established to obtain a homogeneous and stable fine powder, to be then analysed by ICP-MS or ICP-AES after digestion in a high-pressure microwave, and directly by LPAS for leaves. In all cases, particular attention was paid to avoid any contamination from the contact materials used during the pre-treatment procedures. For this purpose, all equipment was thoroughly washed with ultrapure H_2_O (resistivity > 15 MΩ cm) and anti-contamination contact materials (e.g., with ceramic blades) were used. Each sample of drupes was pitted with ceramic knives; the pulp was then crushed and homogenized using a Büchi B-400 mincer with ceramic blades. Finally, the minced product obtained was lyophilized using a Virtis Advantage Plus lyophilizer (freezing of the sample in 3 phases from −10 to −30 degrees, primary drying in 8 phases from −30 to +30 degrees, vacuum from 180 to 50 Pa, total time: 67 h). Each sample of dried olive leaves was ground with an Analysette 3 Pro_fritsch mill (equipped with a grinding head for conversion to Vibratory Micro MIII PULVERISETTE). Each sample was then further homogenised and stored at room temperature until analysis.

### 2.3. Determination of Toxic and Potentially Toxic Elements in Olive Drupes and Leaves

#### 2.3.1. Sample Dissolution

Lyophilised drupes and dried olive leaves were submitted to a dissolution procedure using a Milestone ETHOS-UP high-pressure microwave mineralizer. For both matrices, the procedure was set up in order to obtain a complete dissolution with the minimum final dilution. 

For drupes, accurately weighed sample test-portions of 0.300–0.500 g were treated with 6 mL of HNO_3_ (69%*_V_*_/*V*_) + 4 mL H_2_O and 6 mL of HNO_3_ (69%*_V_*_/*V*_) + 4 mL of H_2_O_2_ with a two-step program at controlled temperature. Complete dissolution was obtained by treating 0.500 g of sample with 6 mL of HNO_3_ (69%*_V_*_/*V*_) + 4 mL H_2_O.

For the olive leaf samples, complete dissolution was obtained by treating 1 g of sample with 6 mL of HNO_3_ (69%*_V_*_/*V*_) + 4 mL H_2_O. The microwave dissolution program is shown in Table 1. 

At the end of the mineralization cycle (30 min), a first 10 min microwave cooling cycle was carried out, and then the containers were cooled and depressurized under a chemical hood. The obtained solutions were quantitatively transferred into volumetric glass flasks and brought to the final volume of 25 mL. All dissolutions were made in triplicate.

Reagent blanks were also prepared by using the same reagents as well as process blanks by applying the same dissolution procedure. All the blanks were then subjected to elemental analysis under the same conditions as the samples.

#### 2.3.2. ICP-MS Analysis of Drupes

Toxic and potentially toxic elements in olive drupes were determined by ICP-MS using a Bruker Aurora M90 (90-degree ion mirror ion optics, Collision Reaction Interface).

The ICP-MS system tuning was preliminarily carried out using a tune solution composed of Be, Mg, Co, In, Ba, Ce, Ti, Pb, and Th, at the concentration of 5 μg/L. The tune solution was prepared to cover a wide range of masses and to optimize sensitivity and resolution. It also allowed obtaining the mass calibration with the control of the accuracy of the mass/charge ratios (*m*/*z*). The plasma parameters were optimized to minimize interferences due to the presence of oxides; the oxide ion levels were checked considering the CeO +/Ce + ratio (<2%) based on the analysis of the tune solution. The double-charged ions were monitored considering the ratio 137Ba ++/137Ba + (<3%) based on the analysis of the tune solution.

In order to evaluate and correct the non-spectroscopic interferences in the method optimization phase, the appropriate dilution of the samples was evaluated so that their total dissolved solids (TDS) % was <0.2%.

The ICP-MS operating conditions were as follows: RF power 1.40 kW; plasma flow rate 18 L/min; auxiliary gas flow rate 1.80 L/min; nebulizer gas flow rate 1.00 L/min; peak hopping scanning mode; integration time 240 s/sample; and dwell time 10,000–60,000 µs.

The isotopes measured were: As75, Cd111, Cd114, Co59, Cr52, Cu65, Ni60, Ni58, Ni64, Pb (206,207,208), Rb85, Rb87, Sb121, Se76, Se78, Sn118, Sr87, Sr88, Ti49, V51, Zn64, Zn66, Zn68, and Zr90.

An external calibration was carried out for all the elements using 5 solutions of the considered elements at known concentration and the blank solution (ultrapure H_2_O acidulated with HNO_3_ 1%*_V_*_/*V*_). The calibration standards were prepared by diluting certified solutions of 1 g/L, brought to volume in graduated flasks (class A) with 1% HNO_3_ acidified ultra-pure water.

For the validation of the method, the limit of detection, the limit of quantification, the linearity range, and the repeatability were evaluated.

The results were expressed in mg/kg as the mean of 3 replicate measurements. For all quantified elements, the RSD% (relative standard deviation) was between 3% and 5%.

#### 2.3.3. ICP-AES Analysis of Olive Leaves

The content of toxic and potentially toxic elements in olive drupes was determined by ICP-AES using a Varian Vista MPX (quartz three-chamber axial torch, air-cooled 40 MHz free running radio frequency generator, plasma, auxiliary and nebulizer gas: argon, glass cyclonic spray chamber, glass concentric flow nebulizer—Meinhardt type, and a CCD detector).

First, the torch alignment and wavelength calibration were performed by reading a Mn standard solution (5 mg/L) and a multi-element standard solution (Al, As, Ba, Cd, Co, Cr, Cu, Mn, Mo, Ni, Pb, Se, Sr, and Zn—5 mg/L; K—50 mg/L), respectively.

To set up the method, preliminary tests were carried out aimed at optimizing the analytical conditions based on the signal-to-background ratio (S/B).

A study of the spectral interferences was then conducted in order to select the analytical wavelengths; where there were no spectral interferences, the most intense wavelength was selected as the analytical one.

The ICP-AES operating conditions and parameters were as follows: Rf power 1.20 kW; plasma flow rate 15 L/min; auxiliary gas flow rate 1.50 L/min; nebulizer gas flow rate 1.10 L/min; pump rate: 30 rpm; internal stabilization delay 10 s; and sample uptake delay 35 s.

Signal stability tests were also carried out by means of replicated measurements of the standard solutions.

The accuracy was evaluated based on the results obtained from the analysis of Certified Reference Materials (CRMs) analysed in parallel with the samples, following the same procedure. In particular, the following CRMs were used: NIST SRM 1570a “Trace Elements in Spinach Leaves” and NIST SRM 1573a “Tomato Leaves”, certified for the content of elements (macro-elements, micro-elements and trace elements). The obtained recovery was around 95% for all the elements.

The results were expressed in mg/kg as the mean of 3 replicate measurements. For all quantified elements, the RSD% was between 3% and 5%.

### 2.4. Reagents and Glassware

In all cases, equipment, disposables, and high-pure reagents and ultra-pure deionized water (resistivity > 15 MΩ cm), specifically designed for ultra-trace analysis, were used. 

All glassware and the microwave Teflon vessels were preliminarily ultra-cleaned with an automatic washing system using acid vapours (concentrated HNO_3_) at reflux (Milestone TraceCLEAN^®^) and then rinsed with ultrapure water (resistivity > 15 MΩ cm).

### 2.5. LPAS Analysis

**Figure 2:**
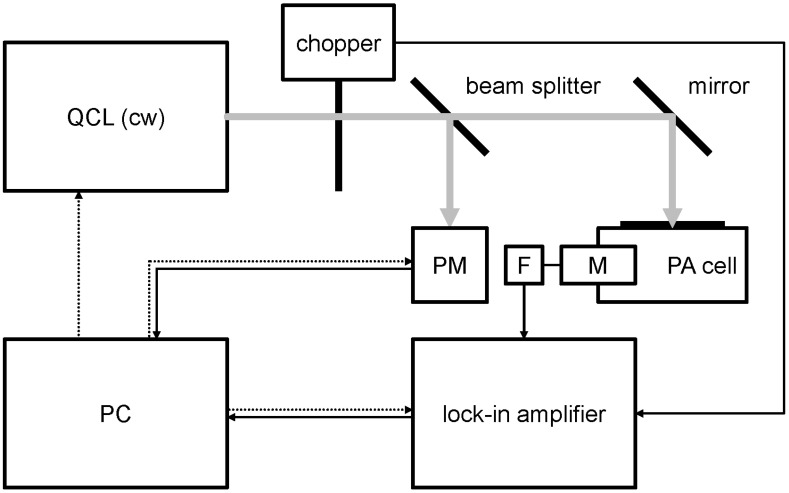
Block diagram of the instrument (cw: continuous wave, F: active low pass filter, M: microphone, PA: photoacoustic, PC: personal computer, PM: power meter, QCL: quantum cascade laser). Grey continuous line: laser beam. Black continuous line: signal. Black dotted line: control.

The LPAS system has been detailed elsewhere [29], and thus only a summary description will be given here; its block diagram is given in Figure 2. The continuous wave (cw) emitted by a quantum cascade laser (QCL) is chopped at an audio frequency and irradiates the sample inside the photoacoustic (PA) cell. The radiation is absorbed by the sample, with the consequent temperature increase, adiabatic expansion, and pressure wave generation. Acoustic resonance amplifies the signal that is detected by the microphone (M) coupled with the lock-in amplifier. A small part of the laser beam is sent to the power meter (PM) by the beam splitter. A personal computer (PC) controls the experiment.

A dedicated software fully manages the spectra acquisition. The user has to input on the interface:

- minimum wavelength, maximum wavelength; and wavelength step;
- number of measurements at a given wavelength (each measurement lasts 1 s);

and simply start the instrument from the PC that records the microphone signal and laser power per measurement. Usually, each point of the LPAS spectrum (photoacoustic signal) is given by the ratio of the average of microphone signals and the average of the laser powers.

### 2.6. Data Analysis and Statistics

The concentration data of toxic and potentially toxic elements were presented in mg kg^−1^ of dry weight of plant parts. Statistical analysis of the data obtained through ICP-MS and ICP-AES analyses was performed using the MATLAB R2015b software. 

To highlight significant differences in the elemental profile of drupes and leaves among the cultivars of the same field, or among the different fields, a one-way ANOVA test was carried out, and when there was a difference between the mean values, Tukey’s test (*p* < 0.05) followed. Pearson’s coefficient was calculated to measure the level of the relationship between the elements. 

Two-way ANOVA was then performed to highlight possible relationships between the levels of individual elements with respect to the experimental field and the cultivar. To examine the ICP-MS, ICP-AES, and the LPAS data, Principal Components Analysis (PCA) was performed. The PCA analysis [30,31] is largely used in exploratory data analysis to obtain preliminary information especially because it is an unsupervised technique. This treatment is applied in order to eliminate data redundancy by changing the number of variables and consequently representing the sample set in a reduced dimension hyperspace [32,33], allowing to evidence statistical differences between the analysed samples. The PCA algorithm finds a number of hypothetical variables called Principal Components (PC), representing as much as possible the variability in multivariate data [34,35]; the new variables are the result of a linear combination of the original variables, where each descriptor has its own “weight”. Generally, according to a descending order of weight, the first new variables are able to represent most of the variation in the original variables [36].

Moreover, for the ICP-MS and ICP-AES data, the correlation among elements, and their influence on the principal components and onto the clusters of different experimental fields, can be evaluated using a biplot whose axes are appropriately scaled to best fit together the data points and vectors of the original variables (i.e., the concentrations of elements), representing the principal components of the model.

The LPAS spectra were first normalized to the maximum peak such that the matrix constructed for the PCA included values between 0 and 1. 

## 3. Results

### 3.1. Elements in Drupes

The results obtained for the elemental total content—determined by ICP-MS—in the different drupes’ cultivars for the two different production areas are shown in Table 2 and Table 3. The tables also report a comparison of the obtained concentration values with the limit value in oil and fat set by Regulation (EC) No.1881/2006 (consolidated version 2021) for Pb. 

For the ENEA production area (Table 2), the most abundant elements in drupes were Sr and Cu, while Pb was the element with the lowest concentration. The concentration level of the elements decreased as follows: Sr, Cu, Rb, Ti, Cr, Ni, V, As, Sn, Zr, Co, and Pb. The results for Sb and Cd were below the detection limit of the analytical technique (0.002 mg/kg and 0.0003 mg/kg, respectively).

For the Allumiere production area (Table 3), the most abundant elements in drupes were Rb, Cu, and Sr, while Pb was the element with the lowest concentration. The concentration level decreased as follows: Rb, Cu, Sr, Ti, Cr, Ni, V, Co, As. Zr, Sn, Sb. The results for Cd were below the detection limit of the analytical technique (0.003 mg/kg, 0.002 mg/kg, 0.002 mg/kg, and 0.0003 mg/kg, respectively) in all samples, and for Pb (0.002 mg/kg) in some sample as well. In any case, the Pb concentration was in all samples below the limit set by Reg. (EC) 1881/2006 (cons.2021), 0.1 mg/kg.

Having five different experimental fields in the same production area of Allumiere, made it possible to compare the behaviour of the different cultivars in different fields.

The one-way ANOVA was used for checking possible differentiation by cultivar. PCA was used as an exploratory technique to highlight correlations and differences in the elemental total contents in the drupes from the Allumiere production area. The variability between the elemental contents in the drupes was determined only by the experimental field (*p* = 0.05) and not by the cultivar.

**Figure 3:**
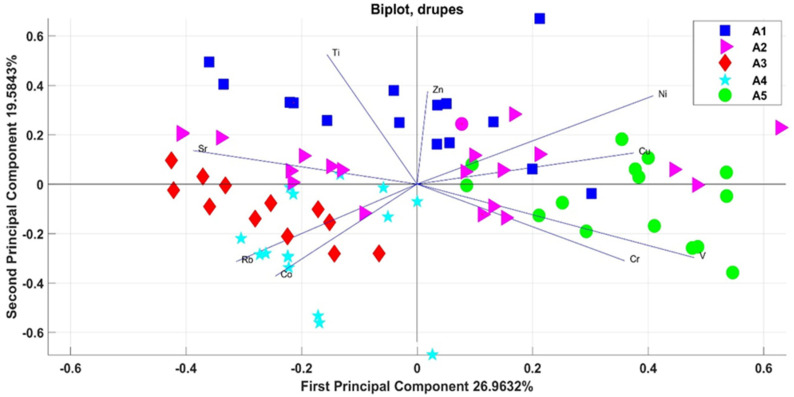
Correlations of the variables (levels of the various elements in drupes) for each statistical unit (field–cultivar) in respect to the main components PC1 and PC2.

The calculation of the two-way ANOVA was used for checking of possible differentiations by cultivar and experimental field. This statistical approach examines the effect of the two factors on the continuous dependent variable. In this case, the correlations of the variables (levels of the various elements) for each statistical unit (field–cultivar) with respect to the main components are graphically shown through a biplot—PCA in Figure 3 (PC1 and PC2). Although the first two main components represented 46% of the total variation in the data set (with eigenvalues of 2.4267 and 1.7626), the bi-dimensional plot showed a separation between the experimental fields.

Mainly, drupe samples from the different experimental fields were clustered in five different groups, as shown in Figure 3. The drupes from field A5 were grouped on the right side (depending on a higher concentration of Cu, V, and Cr), and the drupes from the A3 and A4 fields were grouped in the lower-left square (depending on a higher concentration of Rb and Co). The drupes from field A1 were grouped in the upper squares (depending on a higher concentration of Ti and Zn). So, in the same production area, groupings between the different experimental fields can be noted, although in some cases there is a partial overlap between the fields. 

### 3.2. Elements in Olive Leaves

The results of the elemental total content—determined by ICP-AES—in leaves from the different cultivars and in the two different production areas are shown in Table 4 and Table 5.

The concentration of the elements in leaf samples of the ENEA production area (Table 4) decreased as follow: Al, Sr, Fe, Ba, Rb, Mn, Zn, Cu, and Ti, respectively. The results for As, Sb, Pb, Ni, Cd, Co, Cr, and V were below the detection limit of the analytical technique (6 mg/kg, 5 mg/kg, 1 mg/kg, 0.8 mg/kg, and 0.6 mg/kg, respectively).

The concentration of the elements in leaf samples of the Allumiere production area were variable between the different experimental fields (Table 5).

Generally, for the Allumiere production area (Table 5), the most abundant elements in leaves were Al, Fe, and Rb, while Ti was the element with the lowest concentration. The results for As, Sb, Pb, Cd, Co, Cr, and V were below the detection limit of the analytical technique (6 mg/kg, 5 mg/kg, 1 mg/kg, 0.8 mg/kg, 0.6 mg/kg, 0.6 mg/kg, 0.3 mg/kg, and 0.2 mg/kg, respectively); the results for Ni were above the D.L. (0.8 mg/kg) only for the leaves of the experimental field A5.

As for the drupes, the one-way ANOVA was used for checking of possible differentiation by cultivar. Then, PCA was used to highlight the correlations and differences on the elemental total content in leaves for the Allumiere production area. The variability between the elemental contents in the leaves was determined only by the experimental field (*p* = 0.05) and not by the cultivar.

**Figure 4:**
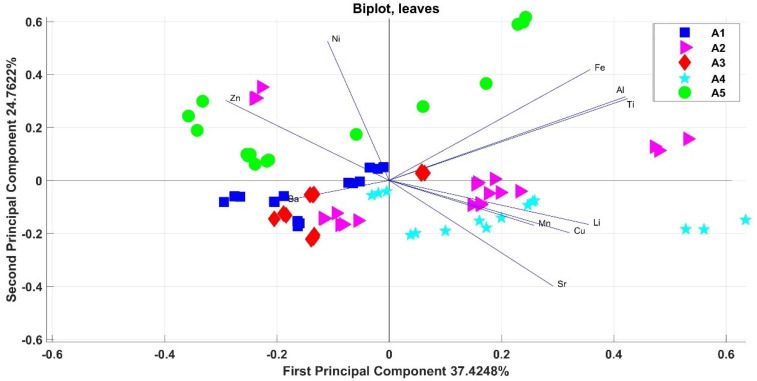
Correlations of the variables (levels of the various elements in leaves) for each statistical unit (field–cultivar) with respect to the main components PC1 and PC2.

Therefore, the calculation of the two-way ANOVA was used to check for possible differentiations of samples by cultivar and experimental field; the two-dimensional PCA score plot generated with the data set of the leaves is shown in Figure 4 (PC1 and PC2). The first two main components represented 62% of the total variation in the data set (with eigenvalues of 3.7425 and 2.4762), and the bi-dimensional plot showed a separation between the samples of the five experimental fields. As can be seen on PC1 (Figure 4), the leaves samples results grouped according to the experimental field. In particular, the samples of the experimental field A1 were grouped in the lower-left square, and the leaves of the experimental field A3, too. In the lower-right square, there was a cluster for all the leaves of field A4 (due to a higher concentration of Cu and Mn) and a certain “proximity” appears with the leaves of the experimental field A2 (where the Mn, Cu, Al, and Ti content was higher than in the leaves from the other fields). Only two cultivars of field A2 differ from this group (Figure 4): Ascolana (which shows a similarity with the leaves of fields A1 and A3) and Uovo di piccione (which shows a similarity with the leaves of field A5, especially for the Zn content). In the upper two squares, the leaves of the A5 field were clustered, while in the right square the cultivars Leccino and Cipresso (in which the content of Cr, Fe, Al, Ti, and V are higher than the other leaves) were clustered; finally, in the left square there was the cluster for the Ascolana, Maurino, and Frantoio cultivars (in which the content of Ni and Zn are higher than the other leaves). Furthermore, in this case, in the same production area, groupings between the different experimental fields can be noted, although in some cases there is a partial overlap between the fields. 

The results on the total content of the different parts of the plants (drupes and leaves) between the two production areas ENEA and Allumiere were then compared (*p* = 0.05), and in order to display the effect of the production area, PCA followed.

The average total content of Cu (an essential element for the plant) in drupes and leaves was comparable in all the cultivars and fields considered. The total content of Cu in leaves of the experimental field A4 was statistically different (level of significance *p* < 0.05) from the other samples of the different experimental fields considered. The total content of Cr in drupes of the Cipresso and Uovo di piccione cultivars of ENEA experimental field and Leccino cultivar of the A5 experimental field was statistically different (*p* < 0.05) from the other samples of drupes considered. The total content of Ni in drupes was very variable between the experimental fields and cultivars; it was statistically different (*p* < 0.05) for the Uovo di piccione and Maurino cultivars of the ENEA experimental field, for the Pendolino cultivar of the A1 field, for the Uovo di piccione cultivar of the A2 field, and for the Cipresso and Ascolana cultivars of the A5 experimental field. The total content of Pb was above the detection limit of the analytical technique for the drupes of the ENEA field (but in all cases at a very low concentration—maximum 0.05 mg/kg). The drupes of the ENEA field, in all cases, had a content of Ti statistically different (*p* < 0.05) from the other experimental field. The total content of V in the drupes was quite variable by cultivar and by field; it was statistically different (*p* < 0.05) for all the samples of ENEA and the A5 fields, for Canino, Frantoio, and Uovo di piccione cultivars of the A1 field, for the Maurino cultivar of the A3 field, and for the Pendolino and Ascolana cultivars of the A4 field.

The results for As and Cr, Ni, Pb, and V were always below the D.L.

**Figure 5:**
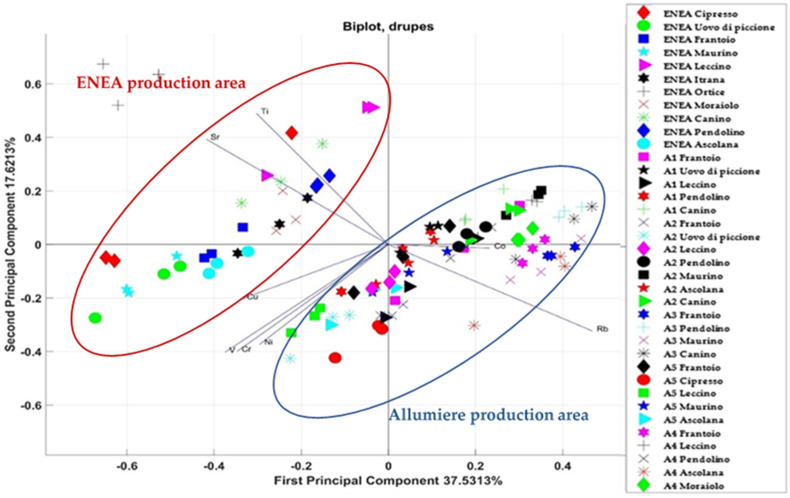
Correlations of the variables (levels of the various elements in drupes from ENEA and Allumiere production areas) for each statistical unit (cultivar) with respect to the main components PC1 and PC2.

Furthermore, in this case, PCA was used to highlight correlations and differences in the elemental total content in leaves and drupes of each production area and each cultivar. The correlations obtained for all the drupes of the different cultivars in the two production areas are shown in the bi-dimensional PCA score plot of Figure 5 (PC1 and PC2). The graph shows the vectors related to the test elements (Sr, Cu, Rb, Ti, Ni, Cr, V, and Co) and the points related to different cultivars (Pendolino, Frantoio, Uovo di piccione, Maurino, Leccino, Canino, and Ascolana) of the two production areas.

Although the first two main components account for 55% of the data variance (with eigenvalues of 3.002 and 1.4097), as shown in Figure 5, the projection of the variables on the PC1 indicated that the drupes from ENEA field were negatively correlated with Ti and Sr, whereas the drupes from the Allumiere fields were positively correlated with Co and Rb and negatively correlated with V, Cr, and Ni. Thus, the drupes from the different production areas formed two different groups: one for the ENEA production area in the left side, and one for the Allumiere production area in the right side. Within the cluster of Allumiere production area, other small group formed by the samples collected at the same experimental field can be identified. In this case, no groupings were found according to the cultivar; therefore, it is possible to cluster only according to the geographic area. 

**Figure 6:**
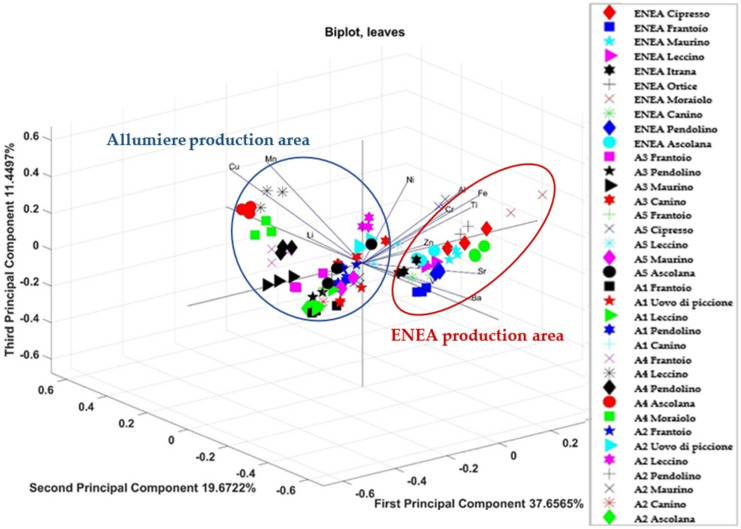
Correlations of the variables (levels of the various elements in leaves from the ENEA and Allumiere production areas) for each statistical unit (cultivar) with respect to the main components PC1, PC2, and PC3.

The correlations obtained for the elemental content in all the leaves of the different cultivars in the two production areas are shown through a tri-dimensional PCA score plot in Figure 6 (PC1, PC2, and PC3). The three main components account for 69% of the data variance (with eigenvalues of 4.5188, 2.3607, and 1.374). The biplot showed the vectors related to the test elements (Cu, Mn, Li, Ni, Zn, Fe, Ti, Al, Sr, and Ba) and the points related to different cultivars of the two production areas. Two distinct groups can be highlighted, one for all the leaves of ENEA production area and one for all the leaves of Allumiere production area. The elements that determined the ENEA cluster were Sr, Ba, Al, Fe, and Ti, while the elements that determined the Allumiere cluster were Cu and Mn. Even for the leaves, there was no grouping depending on the cultivar, while the samples are grouped by geographic area.

### 3.3. LPAS Results for Olive Leaves

**Figure 7:**
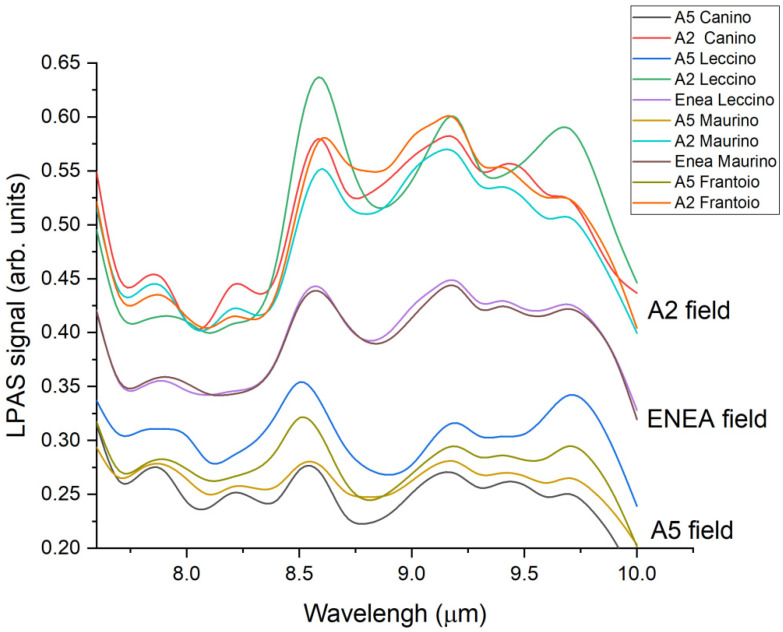
LPAS spectra of the analysed olive leaf cultivars.

In order to assess the potential of the new LPAS analytical technique for determining geographic origin, we selected four representative areas and varieties (among all those studied by elemental analysis) to be investigated with this method. The spectra obtained from LPAS (Figure 7) shows the LPAS signal for each emitted wavelength (μm). The results highlight that the samples were grouped mainly by cultivation areas. In fact, each sample from the A2 (Leccino, Maurino, Frantoio, and Canino cultivars), A5 (Leccino, Maurino, Frantoio, and Canino cultivars), and ENEA (Leccino and Maurino cultivars) areas exhibited similar spectral pattern and intensity emission. These results have confirmed what was already obtained by elemental analysis (Figure 6). Nevertheless, in some cases, such as for A2 Leccino and A2 Canino, specific bands around 9.65 and 8.25 µm, respectively, were observed. 

**Figure 8:**
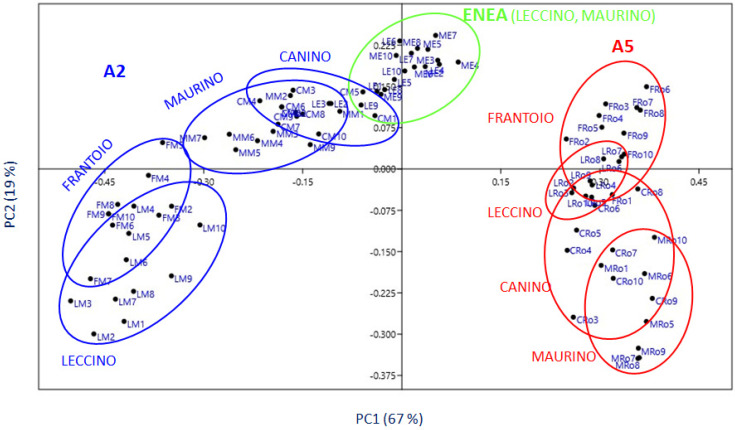
Results of the PCA (LPAS spectra) in the 2D space delimitated by the first two principal components (PC1 and PC2); samples indexed as LM, FM, MM, and CM represent, respectively, the Leccino, Frantoio, Maurino, and Canino cultivars from the A2 field; MRo, CRo, LRo, and FRo samples represent, respectively, the Leccino, Frantoio, Maurino, and Canino from the A5 field; and ME and LE are the Maurino and Leccino samples coming from the ENEA field.

The PCA model was constructed based on the covariance matrix by using the LPAS spectral data (photoacoustic signal for each emitted wavelength) of all the analyzed samples (from the A2, A5, and ENEA fields). The two-dimensional PCA score plot generated with the LPAS data set of the leaves is shown in Figure 8 (PC1 and PC2). The first two PCs were able to explain more than 80% of the LPAS data variability (with eigenvalues of 0.08243 and 0.02381), and the bi-dimensional plot showed a separation between the samples of the three considered experimental fields. It can be seen in Figure 8 that the results for the leaf samples were grouped predominantly according to the experimental field. In particular, the samples of the experimental field A2 were grouped on the left side of the graph, and the leaves of the experimental field A5 on the right side. In the upper squares, there was a cluster for the leaves coming from the ENEA field and a certain “proximity” appears with the leaves of the experimental field A2.

## 4. Discussion

Based on the hypothesis that the mineral content of the soil in which the olives are grown dictates the multi-elemental composition of the resulting fruits [8,10,11,37], the use of ICP-AES and ICP-MS analytical techniques combined with chemometric tools, such as PCA, was evaluated to assess the applicability of these techniques for the determination of the geographical origin of olive products.

In addition, for the same purpose, the suitability of the new analytical technique LPAS was evaluated.

The results obtained for the elemental content in the drupes were compared with the literature, and it might be noted that the values obtained were in good agreement with it [38,39,40]. Moreover, the data obtained indicate that the drupes’ elemental profiles are consistent with those reported in the literature for uncontaminated soils. The contents of Pb, Cd, Cr, Mn, Fe, Ni, and Co in the ENEA drupes are in good agreement with those reported in the literature for plants grown on uncontaminated soils, while the ones from Allumiere are lower [40]. The concentrations of major and trace elements in olives can be used both to establish the geographical origin of olives and, like other authors reported [19,41], as an indicator of soil pollution.

The results obtained for the elemental content in the leaves were compared between the production areas. Cu in leaves samples from A4 field was more concentrated, probably due to the use of the copper fungicide, which serves to prevent some olive diseases such as “peacock’s eye” and “mange” and which is applied on leaves when the plant is resting vegetatively.

The possibility to study different cultivars in the same experimental field and, at the same time, the same cultivars in two different production areas, made it possible to study both the influence of cultivars and the effect of the production area on the multi-elemental content in drupes and leaves.

One-way ANOVA was used for checking of possible differentiations by cultivar for the drupes and leaves of the Allumiere production area. In no case it was possible to distinguish between cultivars with this approach.

Then, two-way ANOVA was used for checking of possible differentiations by cultivar and experimental field (A1, A2, A3, A4, and A5) for both the drupes and leaves in the Allumiere production area. In both cases, PCA highlighted groupings between the different experimental fields, although in some cases there was a partial overlap between the fields. This result can be explained considering that the experimental fields belong to the same production area, i.e., the Allumiere one. In fact, the elemental bioavailability in soils is influenced by several factors, such as soil texture, pH, moisture, and clay complexes, that are characteristic of the production area [42,43]. In order to assess the effect of the production area, the results on the elemental total content in both drupes and leaves from the two production areas (ENEA and Allumiere) were compared. Furthermore, in this case, PCA was used to highlight correlations and differences between production areas and among cultivars. As Figure 5 and Figure 6 show, this approach was effective for distinguishing samples by production area, while in no case there was an overlap between the two groups due to the effect of the cultivars.

The obtained results demonstrated the possibility to discriminate the olive and leaf samples considered in this study according to the production area, but not according to their cultivar. The elemental analysis by ICP-AES and ICP-MS, combined with chemometrics, resulted in a valuable tool to discriminate samples by the production area. Further studies are needed to assess which soil characteristics may influence the presence of elements in olives and olive products, as well as to verify how the elemental bioavailability can be affected by the use of fertilizers or fungicides in order to establish the wider applicability of the proposed method for geographical traceability in different growing conditions.

LPAS was demonstrated as a suitable tool, too, confirming for olive leaves the clustering per geographic area. This represents a very interesting result, particularly taking into account that the clustering was based—for ICP-AES and ICP-MS—on elemental profiles, while for LPAS on molecular profiles. The LPAS technique, being very fast (a few minutes with no need of sample pretreatment, neither with the addition of reagents), can be therefore used alongside the conventional analytical approaches as a useful tool. In particular, it can be proposed as a fast pre-screening technique.

## 5. Conclusions

Olives and olive products are particularly important for the national agro-industrial sector, thus determining the origins of these products is essential to establish their authenticity and to detect any possible commercial fraud. This study showed how elemental analysis and LPAS analysis combined with chemometrics can be a valuable tool for this purpose. The attribution of drupes and leaves to their production area was not hindered by the effect of the cultivar; the production area and therefore the type of soil and its characteristics, along with the pedoclimatic conditions, seem to have a greater impact. In future studies, the elemental profile of the drupes should be related to other soil characteristics, such as the bioavailable fraction and total content of the elements and climatic factors, to further demonstrate the validity of this method for the assessment of the geographical origin.

## Figures and Tables

**Table 1:** Microwave dissolution program.

	Time (min)	E (W)	T1 (°C)	T2 (°C)	P (bar)
1° step	15:00	1500	200	70	120
2° step	15:00	1500	200	70	120

**Table 2:** Elemental total content (mg/kg d.w.) in drupes from the different cultivars—ENEA.

Year	Cultivar	Sr	Cu	Rb	Ti	Cr	Ni	V	As	Sn	Zr	Co	Pb
2019	Cipresso	18.3	12.54	4.89	1.094	0.532	0.352	0.111	0.033	0.033	0.021	0.016	0.013
2019	Pendolino	12.4	12.45	4.67	0.575	0.191	0.228	0.076	0.025	0.002	0.018	0.024	0.009
2019	Itrana	8.9	11.42	4.48	0.800	0.314	0.328	0.110	0.042	0.010	0.013	0.010	0.005
2019	Frantoio	12.9	13.85	5.69	0.581	0.394	0.366	0.122	0.019	0.033	0.024	0.012	0.039
2019	Uovo di piccione	15.0	13.70	5.85	0.675	0.589	0.644	0.126	0.029	0.036	0.019	0.019	0.031
2019	Moraiolo	11.5	9.20	3.36	0.538	0.430	0.120	0.124	0.090	0.013	0.019	0.027	0.050
2019	Maurino	12.4	22.52	6.59	1.148	0.420	0.517	0.118	0.028	0.031	0.016	0.037	0.037
2019	Ortice	11.6	15.38	1.95	5.770	0.325	0.104	0.111	0.045	0.008	0.022	0.032	0.017
2019	Leccino	15.6	9.06	3.33	1.020	0.226	0.242	0.080	0.034	0.015	0.017	0.027	0.040
2019	Canino	15.7	10.34	4.29	0.645	0.213	0.228	0.101	0.056	0.002	0.021	0.012	0.020
2019	Ascolana	8.8	17.37	5.33	0.516	0.358	0.238	0.134	0.017	0.002	0.008	0.011	0.052
	Mean	13.0	13.44	4.58	1.215	0.363	0.306	0.110	0.038	0.017	0.018	0.021	0.028
	Std.dev.	2.9	3.93	1.33	1.528	0.128	0.162	0.018	0.021	0.014	0.005	0.009	0.016
	Median	12.4	12.54	4.67	0.675	0.358	0.242	0.111	0.033	0.013	0.019	0.019	0.031
	Min.	8.8	9.06	1.95	0.516	0.191	0.104	0.076	0.017	0.002	0.008	0.010	0.005
	Max.	18.3	22.52	6.59	5.770	0.589	0.644	0.134	0.090	0.036	0.024	0.037	0.052
D.L. (mg/kg d.w.)		0.0003	0.002	0.001	0.002	0.002	0.0001	0.001	0.002	0.002	0.003	0.001	0.002
Limit value—Reg. (CE) N.1881/2006 s.m.i.—mg/kg										.			0.1

**Table 3:** Elemental total content (mg/kg d.w.) in drupes from the different cultivars—Allumiere.

Fields	Cultivar	Rb	Cu	Sr	Ti	Cr	Ni	V	Co	As	Pb
A1	Frantoio	13.78	9.06	5.72	0.400	0.332	0.204	0.066	0.022	0.031	0.003
A1	Uovo di piccione	9.16	15.15	3.40	0.243	0.193	0.179	0.046	0.024	0.014	<0.002
A1	Leccino	13.73	14.77	4.67	0.254	0.310	0.337	0.079	0.028	0.002	0.004
A1	Pendolino	8.78	12.68	3.94	0.319	0.177	0.406	0.049	0.028	0.023	0.013
A1	Canino	10.22	7.09	4.42	0.434	0.199	0.194	0.071	0.022	0.014	0.003
A2	Frantoio	14.77	12.31	6.65	0.093	0.442	0.261	0.124	0.038	0.002	<0.002
A2	Uovo di piccione	11.02	14.11	4.03	0.139	0.353	0.333	0.133	0.016	0.014	0.018
A2	Leccino	9.89	12.68	5.67	0.119	0.334	0.335	0.104	0.051	0.016	0.003
A2	Pendolino	9.95	11.62	5.56	0.137	0.192	0.336	0.053	0.070	0.007	0.007
A2	Maurino	13.63	8.38	7.26	0.149	0.128	0.127	0.036	0.018	0.002	<0.002
A2	Ascolana	11.34	17.13	4.37	0.188	0.265	0.173	0.069	0.030	0.012	0.018
A2	Canino	11.34	10.25	5.11	0.111	0.191	0.146	0.049	0.039	0.008	<0.002
A3	Frantoio	19.94	12.04	18.93	0.224	0.159	0.097	0.059	0.038	0.007	0.017
A3	Pendolino	17.73	9.02	8.96	0.184	0.096	0.047	0.044	0.015	0.026	<0.002
A3	Maurino	18.10	8.76	32.18	0.138	0.260	0.068	0.092	0.052	0.011	<0.002
A3	Canino	18.28	9.04	9.37	0.147	0.089	0.044	0.040	0.022	0.018	<0.002
A4	Frantoio	17.18	10.71	7.75	0.124	0.188	0.088	0.083	0.060	0.006	0.003
A4	Leccino	10.93	10.95	4.28	0.139	0.143	0.054	0.062	0.051	0.002	0.006
A4	Pendolino	11.00	11.59	4.70	0.172	0.225	0.112	0.089	0.060	0.002	0.005
A4	Ascolana	15.33	10.10	5.56	0.092	0.260	0.063	0.085	0.113	0.008	<0.002
A4	Moraiolo	14.70	10.64	6.53	0.099	0.171	0.076	0.075	0.054	0.002	<0.002
A5	Frantoio	8.65	10.10	2.60	0.121	0.302	0.270	0.079	0.001	0.020	0.003
A5	Cipresso	14.88	13.82	4.83	0.129	0.314	0.613	0.114	0.016	0.020	0.004
A5	leccino	8.72	12.68	2.88	0.102	0.573	0.119	0.156	0.014	0.010	0.032
A5	Maurino	10.84	8.79	3.29	0.114	0.290	0.301	0.107	0.002	0.013	<0.002
A5	Ascolana	10.50	11.76	2.47	0.125	0.238	0.598	0.099	<0.001	0.027	0.007
	Mean	12.86	11.36	6.74	0.173	0.247	0.215	0.079	0.035	0.012	0.009
	Std.dev.	3.37	2.38	6.11	0.090	0.108	0.158	0.031	0.025	0.008	0.008
	Median	11.34	11.27	4.97	0.139	0.232	0.176	0.077	0.028	0.011	0.006
	Min.	8.65	7.09	2.47	0.092	0.089	0.044	0.036	0.001	0.002	0.003
	Max.	19.94	17.13	32.18	0.434	0.573	0.613	0.156	0.113	0.031	0.032
D.L. (mg/kg d.w.)		0.001	0.002	0.0003	0.002	0.002	0.0001	0.001	0.001	0.002	0.002

**Table 4:** Elemental total content (mg/kg d.w.) in leaves from the different cultivars—ENEA.

Year	Cultivar	Al	Sr	Fe	Ba	Rb	Mn	Zn	Cu	Ti
2019	Cipresso	127	225	70.7	58.5	77	20.13	13.8	6.9	3.1
2019	Pendolino	89	157	60.4	188.8	117	17.08	12.5	5.3	2.2
2019	Itrana	81	117	57.4	92.5	183	16.71	9.6	5.7	2.0
2019	Frantoio	69	189	46.3	284.3	168	19.18	15.8	4.7	1.5
2019	Uovo di piccione	116	203	67.1	250.7	65	19.14	9.8	4.7	2.6
2019	Moraiolo	170	232	93.3	43.3	75	22.13	13.3	4.9	4.4
2019	Maurino	108	166	64.3	169.6	160	18.30	11.2	5.8	2.5
2019	Ortice	124	284	77.9	98.6	110	25.81	9.9	4.9	2.9
2019	Leccino	88	261	61.4	117.6	164	22.76	9.5	4.2	2.2
2019	Canino	93	167	55.7	194.6	151	17.77	11.9	5.3	2.3
2019	Ascolana	100	160	59.5	96.1	117	21.76	12.2	5.1	2.5
	Mean	106	196	64.9	145.0	126.1	20.07	11.8	5.2	2.6
	Std.dev.	28	50	12.5	78.4	41.7	2.78	2.0	0.7	0.7
	Median	100	189	61.4	117.6	117.1	19.18	11.9	5.1	2.5
	Min.	69	117	46.3	43.3	64.6	16.71	9.5	4.2	1.5
	Max.	170	284	93.3	284.3	182.7	25.81	15.8	6.9	4.4
	D.L. (mg/kg d.w.)	5	5	0.2	0.1	5	0.04	0.1	0.4	0.5

**Table 5:** Elemental total content (mg/kg d.w.) in leaves from the different cultivars—Allumiere.

Fields	Cultivar	Al	Sr	Fe	Ba	Rb	Mn	Zn	Cu	Ti
A1	Frantoio	36	34	29.3	9.8	23	6.17	8.5	5.4	0.8
A1	Uovo di piccione	53	32	32.1	20.0	19	8.60	12.2	6.6	1.1
A1	Leccino	31	26	27.0	8.2	23	11.20	16.6	7.2	0.6
A1	Pendolino	62	35	41.4	13.0	43	14.27	15.4	10.4	1.2
A1	Canino	72	35	45.0	11.8	49	17.68	19.2	7.7	1.4
A2	Frantoio	84	43	44.7	5.8	<5	20.53	7.7	10.4	1.7
A2	Uovo di piccione	54	13	45.3	8.0	<5	21.14	22.7	10.4	1.1
A2	Leccino	134	35	64.1	5.3	<5	26.59	6.2	29.3	2.4
A2	Pendolino	61	45	48.8	7.9	94	27.04	9.6	15.2	1.4
A2	Maurino	78	33	44.7	4.3	88	12.70	8.1	14.0	1.7
A2	Ascolana	34	31	32.4	3.4	68	14.92	9.1	9.9	0.8
A2	Canino	36	41	33.5	6.3	80	13.32	12.4	7.2	0.9
A3	Frantoio	30	27	26.1	5.3	72	30.08	11.4	4.8	0.7
A3	Pendolino	50	24	33.5	6.1	62	19.24	10.3	3.9	0.9
A3	Maurino	27	33	29.2	6.4	58	39.65	9.2	4.1	0.6
A3	Canino	78	30	45.9	7.1	70	29.27	10.4	4.1	1.4
A4	Frantoio	49	35	32.2	6.2	<5	27.97	8.9	49.9	1.0
A4	Leccino	92	47	49.8	4.6	<5	36.75	7.2	74.5	1.9
A4	Pendolino	49	21	38.0	2.0	<5	32.53	13.3	48.5	1.0
A4	Ascolana	72	26	42.3	2.7	<5	47.77	7.8	77.5	1.6
A4	Moraiolo	66	44	38.1	7.1	<5	38.67	12.6	64.7	1.3
A5	Frantoio	43	12	34.6	7.2	18	14.92	13.8	6.8	0.8
A5	Cipresso	117	21	82.0	3.8	10	18.01	12.8	5.0	2.3
A5	Leccino	88	13	51.8	2.1	28	15.18	13.5	8.6	2.0
A5	Maurino	52	17	35.4	15.0	26	12.07	11.1	4.6	1.0
A5	Ascolana	39	8	27.2	4.0	7	16.91	18.7	5.9	0.9
	Mean	61	29	40.5	7.1	48	22.04	11.9	19.1	1.2
	Std.dev.	27	11	12.4	4.1	28	10.71	4.1	23.1	0.5
	Median	53	31	38.1	6.3	49	18.63	11.2	8.2	1.1
	Min.	27	8	26.1	2.0	7	6.17	6.2	3.9	0.6
	Max.	134	47	82.0	20.0	94	47.77	22.7	77.5	2.4
D.L. (mg/kg d.w.)		5	5	0.2	0.1	5	0.04	0.1	0.4	0.5

## Data Availability

Data is contained within the article.
